# Influence of temperature on the cardiac action of dobutamine and levosimendan in isolated perfused hearts

**DOI:** 10.1186/cc12167

**Published:** 2013-03-19

**Authors:** U Aldenhoff, R Vicenzi-Moser, M Vicenzi, D Konrad, E Schwarzl, W Toller

**Affiliations:** 1 Medical University Graz, Austria

## Introduction

Inotropic agents (catecholamines or calcium-sensitizers) are frequently used in hypothermic as well as hyperthermic patient conditions. Divergent results from animal experiments raise doubt as to whether they act to the same extent at different body temperatures. Thus, we studied the influence of clinically relevant temperatures on the hemodynamic effects of dobutamine and levosimendan using the Langendorff model of isolated perfused hearts.

## Methods

Isolated guinea pig hearts (*n = *60) were perfused with incremental doses (10 nM to 10 μM) of dobutamine or levosimendan either at normothermic (37°C), hyperthermic (40°C) or hypothermic (34°C) perfusion conditions. Contractility (+dLVP/dt), relaxation (-dLVP/dt), left ventricular pressures and heart rate were recorded. Data with increasing drug dosage were calculated in percent from baseline for each temperature tested. Data for each drug were analysed by two-way ANOVA for repeated measures including the two main effects of temperature and drug dose and their interaction. Data are reported as mean ± standard deviation.

## Results

The positive inotropic action of dobutamine was least at 37°C, more pronounced at 40°C and best at 34°C (37°C vs. 40°C vs. 34°C: 300 nM 280 ± 71 vs. 301 ± 94 vs. 345 ± 99%, 1 μM 310 ± 74 vs. 327 ± 92 vs. 389 ± 144%, 10 μM 297 ± 69 vs. 339 ± 96 vs. 359 ± 175%; *P <*0.05). Dobutamine's positive lusitropic effect was not significantly altered by temperature. The positive inotropic action of levosimendan was best at 37°C, in hyperthermia and hypothermia only the three highest doses of levosimendan increased contractility (37°C vs. 40°C vs. 34°C: 100 nM 121 ± 21 vs. 134 ± 15 vs. 87 ± 12%, 300 nM 135 ± 22 vs. 137 ± 17 vs. 94 ± 15%, 1 μM 153 ± 22 vs. 147 ± 21 vs. 108 ± 21%, 3 μM 172 ± 19 vs. 150 ± 24 vs. 115 ± 23%, 10 μM 173 ± 31 vs. 156 ± 28 vs. 117 ± 28%; *P <*0.05). The positive lusitropic effect of levosimendan at 37°C was almost absent in hypothermia and hyperthermia (*P <*0.05).

## Conclusion

In isolated perfused hearts, dobutamine has its best positive inotropic effect in hypothermia whereas levosimendan increases contractility best at normothermic conditions. Clinical studies are necessary to confirm these experimental results.

